# Supplementary material for: A UMLS-based spell checker for natural language processing in vaccine safety
Source: BMC Med Inform Decis Mak. 2007 Feb 12;7:3. doi: 10.1186/1472-6947-7-3 (PMC1805499; doi:10.1186/1472-6947-7-3)
Supplement: Additional file 13 — RAP application directory. Sets up directory for RAP (RDF application for PHP) source code [file 1472-6947-7-3-S13.gz › rap/doc/phpdoc/resModel/ResIterator.html]

xml version="1.0" encoding="iso-8859-1"?


Docs For Class ResIterator


## Class ResIterator

Description

Description |
Methods (details)

Implementation of a resource iterator.

This Iterator should be used in a for-loop like: $it = $ontClass->listInstances(); for ($it->rewind(); $it->valid(); $it->next()) { $currentResource=$it->current(); };

History: <LI>10-07-2004 : First version of this class.</LI>

- access: public
- author: Daniel Westphal <at d-westphal dot de mail at d-westphal dot de>
- version: V0.9.1

Located in /resModel/ResIterator.php (line 31)

Method Summary

Description |
Methods (details)

ResIterator
ResIterator
(object ResResource $subject, object ResResource $predicate, object ResResource $object, string $getSPO, object ResModel &$associatedModel, [string $returnType = false])

mixed
current
()

integer
key
()

void
next
()

void
rewind
()

void
setFindLiteralWithLang
(string $language)

boolean
valid
()

Methods

Description |
Methods (details)

Constructor ResIterator (line 127)

Constructor.

$subject, $predicate, and $object are used like inf find(). $getSPO supports the strings 's', 'p', and 'o' to return either the subject, predicate, or object of the result statements. $returnType supports the strings 'ResProperty', 'ResLiteral', 'OntProperty', 'OntClass', and 'Individual' and returns the resources as the matching type.

- access: public

ResIterator

ResIterator
(object ResResource $subject, object ResResource $predicate, object ResResource $object, string $getSPO, object ResModel &$associatedModel, [string $returnType = false])

- object ResResource
  $subject
- object ResResource
  $predicate
- object ResResource
  $object
- string
  $getSPO
- object ResModel
  $associatedModel
- string
  $returnType

current (line 177)

Returns the current item

- access: public

mixed

current
()

key (line 240)

Returns the key of the current item

- access: public

integer

key
()

next (line 165)

Moves Iterator to the next item in the list

- access: public

void

next
()

rewind (line 143)

Resets iterator list to start

- access: public

void

rewind
()

setFindLiteralWithLang (line 252)

Sets that only Literals with the matching language should be returned

- access: public

void

setFindLiteralWithLang
(string $language)

- string
  $language

valid (line 155)

Says if there are additional items left in the list

- access: public

boolean

valid
()

Documentation generated on Thu, 7 Jul 2005 13:43:33 +0200 by phpDocumentor 1.3.0RC3
